# Aging and low-intensity exercise change oxidative biomarkers in brain regions and radiographic measures of femur of Wistar rats

**DOI:** 10.1590/1414-431X20209237

**Published:** 2020-05-08

**Authors:** E.M.S. Silveira, M.C.Q. Santos, T.C.B. da Silva, F.B.O. Silva, C.V. Machado, L. Elias, A. Kolberg, A. Kroth, W.A. Partata

**Affiliations:** 1 Laboratório de Neurobiologia Comparada, Departamento de Fisiologia, Instituto de Ciências Básicas da Saúde, Universidade Federal do Rio Grande do Sul, Porto Alegre, RS, Brasil; 2 Graduada em Medicina Veterinária, Fundação Educacional Dom André Arcoverde (UNIFAA), Centro Universitário de Valença, Valença, RJ, Brasil; 3 Área Ciências da Vida, Universidade do Oeste de Santa Catarina, Joaçaba, SC, Brasil

**Keywords:** Total thiol content, Lipid hydroperoxide levels, Total antioxidant capacity, Superoxide dismutase activity, Treadmill

## Abstract

We investigated changes in oxidative biomarkers in brain regions such as brainstem, cerebellum, and cerebral cortex of 3-, 6-, 18-, 24-, and 30-month-old rats. We also assessed the effects of low-intensity exercise on these biomarkers in these regions of 6-, 18-, and 24-month-old rats that started exercise on a treadmill at 3, 15, and 21 months of age, respectively. Radiographic images of the femur were taken for all rats. A total of 25 rats (age: twelve 6-, ten 18-, ten 24-, and three 30-month-old rats) were used. Lipid hydroperoxide levels increased in cerebellum at 18 months. Total antioxidant activity exhibited lowest values in brainstem at 3 months. Superoxide dismutase activity did not exhibit significant changes during aging. Total thiol content exhibited lowest values in brain regions of 24- and 30-month-old rats. Exercise reduced total thiol content in brainstem at 6 months, but no change occurred in other regions and other ages. Femur increased its length and width and cortical thickness with advancing age. No change occurred in medullary width. Radiolucency increased and sclerosis was found in cortical and medullary bone with advancing age. Exercise reduced radiolucency and medullary sclerosis. Therefore, aging differentially changed oxidative biomarkers in different brain regions and radiographic measures of the femur. Low-intensity exercise only ameliorated some radiographic measurements of femur. Since the present study possessed limitations (small number of rats per group), a beneficial effect of regular low-intensity exercise on oxidative markers in brain cannot be ruled out.

## Introduction

The world population is progressively getting older. The number of older people aged 60 or more will grow from 962 million to nearly 2.1 billion by 2050 (1,2). In Brazil, it is estimated that there will be 64 million seniors by 2050 (2). Given the increasing number of older adults worldwide, understanding aging-induced changes is imperative to promote healthy aging.

Studies have demonstrated that aging is a complex event characterized by a gradual decline of physiological functions and increased susceptibility to disease (3). The brain is a target of the aging process like other cell systems of the body (4). Evidence has shown that oxidative stress is one of the most important factors contributing to biochemical impairments of the brain during aging (3). Oxidative stress may be defined as an excessive amount of reactive oxygen species (ROS), which is the net result of an imbalance between production and destruction of these species (5). ROS include singlet oxygen, superoxide radicals, hydroxyl radicals, and hydrogen peroxide (5). However, numerous endogenous antioxidant systems are in place to ensure that ROS are properly reduced. Among diverse antioxidant systems is superoxide dismutase (SOD), the enzyme that converts the superoxide anion to H_2_O_2_ and thiols (5). The thiol moiety is particularly sensitive to redox reactions and is an established redox sensor that interacts with a variety of oxidants to form in many cases a reversible covalent modification that includes disulfide bond formation, a major mechanism by which protein function can be controlled (6).

The vulnerability to the oxidative stress-associated aging process appears to differ in different brain regions (7). Mitochondria isolated from aged rat cortex and hippocampus showed increased ROS production compared to young rats, which was not observed in mitochondria isolated from the cerebellum (8). Aging also decreased glutathione/oxidized glutathione ratio and increased lipid peroxidation levels and superoxide dismutase activity in cerebral cortex of 24-month-old Wistar rats compared to 3-month-old rats (9). Also, aging increased lipid peroxides and lipid hydroperoxides but decreased SOD activity in the cerebrum, cerebellum, and brainstem of 6-, 13-, 19-, and 26-month-old male albino rats compared to 3-month-old rats (10). However, the research did not assess total thiol content. In mice, a significant increasing pattern of glutathione content was found in the cerebellum and brainstem with age (11). Thus, it is interesting to assess the changes in age-induced total thiol content in different regions of the Wistar rat brain, a common experimental model in studies of aging. Since Wistar rats' median survival age is 29–30 months (12,13) and only a few of the neurochemical changes present at the age of 30 months were found in a group of rats of intermediate age (14), it appears interesting to determine the changes in oxidative parameters in 30-month-old rats.

One strategy that has beneficial effects on aging-induced changes is regular physical activity (3,15). Exercise appears to protect the brain from oxidant action (16). Nevertheless, the effect appears related to training duration and intensity (15,16). Recent research showed that low-intensity exercise ameliorated performance of aged rats in the beam balance test, a test that assesses simple (static) equilibrium (17). However, the effects of low-intensity exercise on changes in oxidative stress biomarkers in different regions of the brain remain unknown, especially in regions with important roles in motor control.

In order to gain insight into these issues, the aim of the present study was to investigate the effects of age and low-intensity exercise on oxidative biomarkers in cerebral cortex, cerebellum, and brainstem, brain regions that have important roles in motor control and equilibrium. Because changes in the nervous system may compromise areas of extreme importance for the mobility of individuals, for example, the musculoskeletal system that includes bones and a network of tendons, ligaments, cartilage, and attached muscles (3), we also took X-ray images of the femur to investigate aging- and exercise-induced changes in this bone.

## Material and Methods

### Experimental animals

All procedures involving animals were approved by the Ethics Committee for Animal Experimentation of the Universidade Federal do Rio Grande do Sul (CEUA-UFRGS #29386). All efforts were made to minimize animal suffering and reduce the number of animals used. To characterize the effect of aging on brain biomarkers, 47 intact 3-month-old male Wistar rats were obtained from a colony maintained by the Universidade Federal do Rio Grande do Sul. In the laboratory, the rats were housed in groups of three per conventional cage, maintained at 22±2°C, exposed to a 12-h light/dark photoperiod, and provided free access to food and water, as previously described by Silveira et al. (17). Briefly, six (n=6) of the rats were acclimated for one week and euthanized by decapitation. The other rat groups were maintained under laboratory conditions until the ages of 6 (n=12), 18 (n=12), 24 (n=12), and 30 (n=5) months. All rats were kept under close observation and regularly weighed, including prior to sacrifice. Since some rats died of natural causes, the final number was ten for 18- and 24-month-old rats, and three for 30-month-old rats.

The animals were classified as young adult (3 months), adult (6 months), middle-aged (18 months), young elderly (24 months), and old (30 months) (17). These ages were chosen because they correspond to human ages of 18, 45, 60, and 75 years, respectively (17,18).

### Exercise protocol

To characterize the effect of regular low-intensity exercise on brain oxidative biomarkers, rats were randomly selected for an exercise program at ages of 3, 15, and 21 months. The exercise program was carried out for 12 weeks (17). Thus, rats at ages 3, 15, and 21 months exercised until ages of 6, 18, and 24 months, respectively. Non-exercised rats remained in their cages (sedentary). Thirty-month-old rats were not exercised because their total number was three at 27 months.

The exercise consisted of running on a motor driven treadmill (running platform: 113×33 cm; Runner, Brazil) for rats. Prior to initiating the exercise program, the exercise groups were familiarized with a treadmill for one week, 3 times/week, 30 min/day. Having successfully performed the exercise protocol the rats began the exercise program. The exercising routine consisted of running on a treadmill for 30 min/day, 3 times/week, for 12 weeks. Each session included a warm-up period of 5 min at 2 m/min, then exercise for 5 min at 5 m/min and 20 min at 8 m/min, while the incline was maintained at 0% (17). All rats performed this exercise protocol when the treadmill was available to them.

The exercise was performed only three times a week to avoid chronic stress, inflammation, or muscle damage, and to allow the liver and muscle to recover glycogen; rats received no stimulation (aversive or appetitive) to motivate them to run (19). All rats were sacrificed at the same time of day (beginning at 8:00 am), without fasting.

### Sample preparation

Rats were sacrificed by decapitation and whole brain and right femur were promptly collected. The femur was carefully released of soft tissue (muscles, ligaments, tendons, nerves, vessels), cooled, and used for X-ray analysis. All cerebral cortex, cerebellum, and brainstem were excised carefully on ice. These tissues were homogenized in 1.15% KCl diluted 1:5 (w/v) containing 1 mM phenylmethylsulfonyl fluoride, and centrifuged at 1000 *g* for 20 min at 4°C. The supernatant was used for assays of total antioxidant capacity (TAC), total thiols content, superoxide dismutase (SOD) activity, and lipid hydroperoxide levels.

### Determination of lipid hydroperoxide levels

The lipid hydroperoxide levels were measured by oxidation of Fe^2+^ by LOOH in an acid medium containing xylenol orange dye, which forms a complex with Fe^3+^, as described by Jiang et al. (20). Briefly, a working reagent was prepared with 81% (vol/vol) of 90% methanol, 2 mM xylenol orange (o-cresolsulfonphthalein-3'-3"-bis [methyliminodiacetic acid sodium salt]) to a final concentration of 100 μM, 1 mol/L sulfuric acid to a final concentration of 25, 40 mM 2,6-di-tert-butyl-4-methylphenol to a final concentration of 4, and 10 mM ferrous sulfate to a final concentration of 250 μmol. Samples were prediluted 1:10 in Milli-Q (Direct-Q3, Millipore SAS, France) water before the test. Then, they were divided into 2 tubes with 90 μL of the sample in each. The samples were incubated for 30 min with 10 μL of 90% methanol or 10 μL of 1 mM triphenylphosphine (TPP). After incubation, the samples were pipetted in duplicate into the microplate and incubated with working reagent (1:9) with stirring for 1 h at room temperature. Absorbance at 560 nm was obtained (spectrophotometer, Zenyth 200rt; Anthos), and the absorbance values of the duplicates obtained with TPP were subtracted from the values for the duplicates without TPP. The assay was replicated 3 times. Results are reported as nmol/g tissue.

### Determination of TAC

TAC was determined with 2,2-azinobis-(3-ethylbenzothiazoline-6-sulfonic acid) radical cation, which, in an acid medium, is decolorized by antioxidants, according to their concentration and antioxidant capacity (21). Briefly, two working reagents were prepared: reagent 1 (acetate buffer 0.4 mol/L, pH 5.8, adjusted using 0.4 M acetic acid solution) and reagent 2 (ABTS^•+^ in acetate buffer 30 mM, pH 3.6). Reagent 2 was prepared as follows: 2.46 g of sodium acetate was dissolved in 100 mL of deionized water (final concentration: 30 mM). Glacial acetic acid (1.705 mL) was diluted to 1000 mL with deionized water (final concentration: 30 mM). The sodium acetate solution (75 mL) was mixed with 925 mL of acetic acid solution under pH meter; the pH of the acetic acid-sodium acetate buffer was 3.6. Then, 278 µL of commercial hydrogen peroxide solution (35%) was diluted to 1000 mL with the buffer solution (final concentration: 2 mM), and 0.549 g ABTS was dissolved in 100 mL of prepared solution (final concentration: 10 mM). After 1 h of incubation at room temperature, the characteristic color of ABTS^•+^ appeared. In assay, reagent 1 was used in a volume of 200 µL, while the volume of sample and reagent 2 was 5 and 20 µL, respectively. The first absorbance of the assay was taken before the mixing of reagent 1 and reagent 2 (as sample blank) and the last absorbance was taken at the end of the incubation period (5 min after the mixing). The assay was replicated 3 times. Results are reported in µM·eq trolox^−1^·g tissue^-1^.

### Determination of total thiol levels

Total thiol content was determined as described by Aksenov and Markesbery (22). Briefly, 30 μL of a tissue sample was mixed with 1 mL of phosphate/EDTA buffer, pH 7.5, and 5,5'-ditiobis (2-nitrobenzoic) acid (DTNB, 10 mM). Control samples, which did not include DTNB, were run simultaneously. The absorbance was read at 412 nm after 30 min of incubation at room temperature. The assay was replicated 3 times. Results are reported as mmol/g tissue.

### Determination of SOD activity

SOD activity was measured based on its action to neutralize superoxide radicals to prevent oxidation of adrenalin to adrenochrome, a colored byproduct that can be measured at 480 nm. The reaction medium contained glycine buffer (50 mM, pH 11.3) and adrenalin (60 mM, pH 2.0). The assay was replicated 3 times. Results are reported as units/mg protein (23).

### X-ray analysis of femur

Since age modifies bone mineral density, cross-sectional area, and strength at different skeletal sites in male Wistar rats, including femur (24), we used X-ray analysis to expand this knowledge. The total number of bones used was 4 for 24-month-old rats and 3 for other ages. The reduction allowed the femur bones to be placed in a single radiographic image.

**Figure 1:**
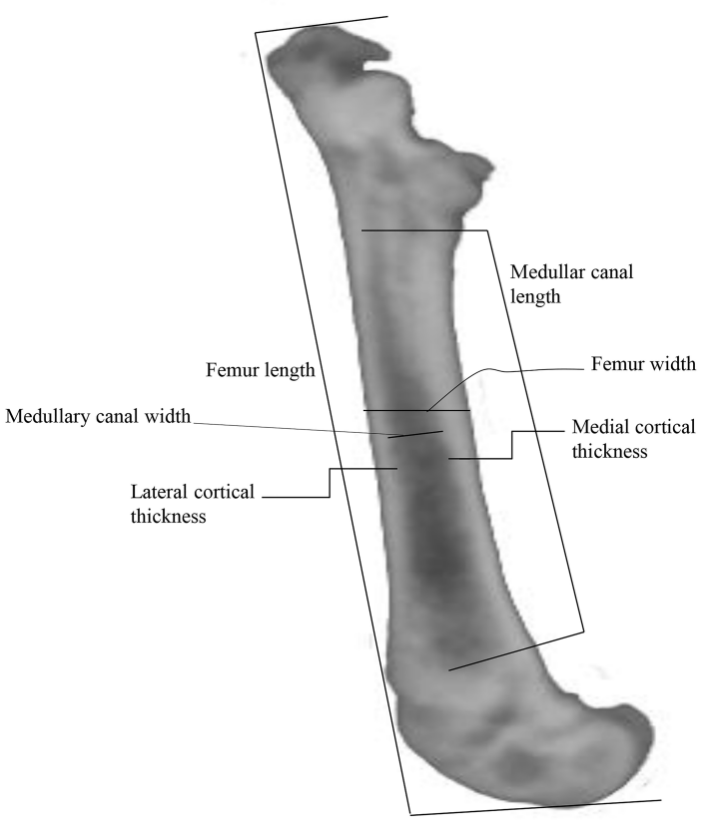
Radiographic lateral view of the femoral bone of a Wistar rat showing parameters measured in this study.

For X-ray analysis, the rat femur specimens were positioned along the long axis. Dorsal-ventral and medial-lateral view X-rays were collected using an X-ray unit (IRay Technology, Model Mars 1417.PSI, 100 mA, 45 kV, 0.01s, China). The radiographs were evaluated using I-Ray Vet software (I-Ray Technology). The following parameters were used for the evaluation of the radiograph images: femur length and width, thickness of cortical bone in its medial and lateral regions, total cortical thickness (medial plus lateral thickness), and medullary width (Figure 1). Radiolucency, radiopacity, remodeling of femoral neck, the presence of sclerosis in cortical and/or medullar, bone deformity, fracture, and presence of osteophyte were also analyzed.

Measurements were always taken from femoral head to femoral condyles. The measures of cortical, medullary, and width were always taken in the diaphysis, in mid-portion of the femur. Radiographs were evaluated by a veterinarian with training and experience in veterinary radiology. The veterinarian was blinded to experimental groups.

### Statistical analysis

The sample size for the present study was based on previous studies using Win Pepi software version 9.1, a significance level of 0.05 and 95% power, which resulted in a sample size of eight rats per group, as described in Silveira et al. (17). However, some rats died during aging, so the final sample size was 5 or 6 rats per group, except for 30-month-old rats which had a sample size of 3 rats. Data from sedentary rats were compared between different ages to assess the effect of aging. Data from exercise rats were compared to sedentary rats to assess the effect of regular low-intensity exercise on rats at different ages. Statistical analyses were performed using SigmaPlot version 11.0 (Systat Software Inc., USA) for Windows. Normal Gaussian distribution of the data was analyzed by the Shapiro-Wilk test, while Levene's test was used to analyze homogeneity of variance. Data of aging for oxidative markers were analyzed using two-way ANOVA, while the effect of exercise was analyzed using three-way ANOVA. Pearson correlation coefficient was used to analyze the correlation between total thiol content and TAC. Data of aging for femur were analyzed by one-way ANOVA, while the effect of exercise was analyzed by two-way ANOVA. The Tukey test was used as the *post hoc* test. Differences were considered statistically significant when P was <0.05. All data are reported as means±SE of the mean.

## Results

### Lipid hydroperoxide levels

**Figure 2:**
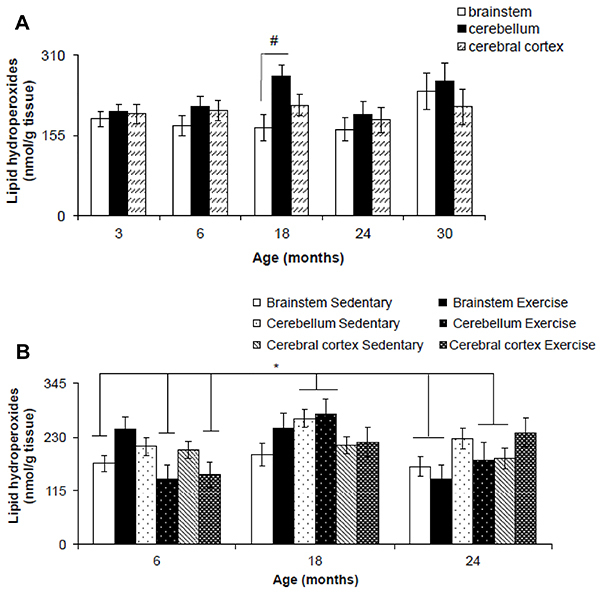
Lipid hydroperoxide levels in the brainstem, cerebellum, and cerebral cortex of Wistar rats of different ages (**A**) and after 12 weeks of treadmill running (**B**). Data are reported as means±SE. ^#^P<0.05, two-way ANOVA; *P<0.05, three-way ANOVA.

A significant difference was found in lipid hydroperoxide levels between brain region (F_(2,76)_=3.478, P=0.034) but not age (F_(2,76)_=1.843, P=0.126). No significant interaction was found between the factors brain region and age (F_(2,76)_=0.845, P=0.565). Lipid hydroperoxide levels were higher in the cerebellum compared to brainstem at 18 months (P=0.006) (Figure 2A).

A significant difference was found in lipid hydroperoxides for age (F_(2,62)_=7.278, P=0.001) but not brain region (F_(2,62)_=0.424, P=0.656) and exercise (F_(2,62)_=0.255, P=0.615). A significant interaction was found between factors brain region and age (F_(2,62)_=3.157, P=0.018). However, there was no significant interaction between factors brain region and exercise (F_(2,62)_=1.531, P=0.223) and exercise and age (F_(2,62)_=1.074, P=0.347). Also, there was no significant interaction between factors brain region, exercise, and age (F_(2,62)_=2.478, P=0.051). *Post hoc* test revealed a significant increase in lipid hydroperoxide levels of the cerebellum of exercise and sedentary 18-month-old rats compared to brainstem of sedentary 6- and 24-month-old rats (P=0.005), cerebellum and cerebral cortex of exercise 6-month-old rats (P=0.041), brainstem of exercise 24-month-old rats (P=0.008), and cerebral cortex of sedentary 24-month-old rats (P=0.005) (Figure 2B).

### SOD activity

**Figure 3:**
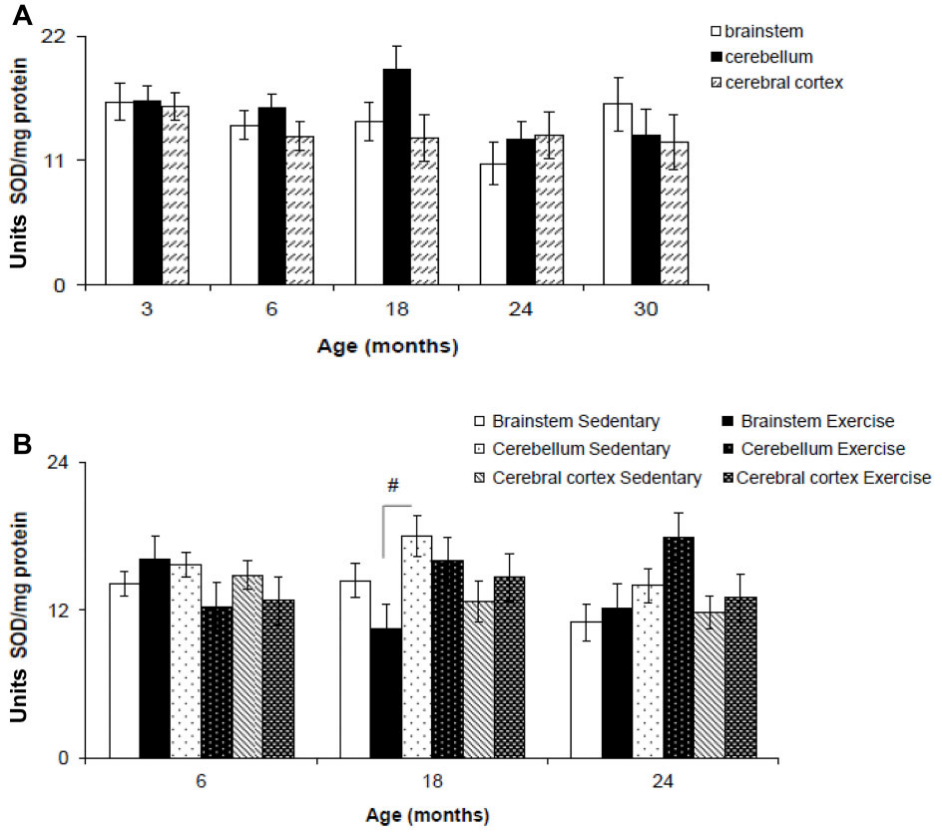
Superoxide dismutase activity (SOD) in the brainstem, cerebellum, and cerebral cortex of Wistar rats of different ages (**A**) and after 12 weeks of treadmill running (**B**). Data are reported as means±SE. ^#^P<0.05, three-way ANOVA.

For SOD activity, two-way ANOVA revealed no significant difference between age (F_(2,76)_=2.232, P=0.072) and brain region (F_(2,76)_=1.450, P=0.240). No significant interaction was found between the factors brain region and age (F_(2,76)_=0.794, P=0.609) (Figure 3A).

A significant difference was found for brain region (F_(2,62)_=4.506, P=0.014) but not for age (F_(2,62)_=0.771, P=0.466) and exercise (F_(2,62)_=0.028, P=0.867) (Figure 3B). No significant interaction was found between factors brain region and age (F_(2,62)_=2.102, P=0.090), brain region and exercise (F_(2,62)_=0.120, P=0.887), age and exercise (F_(2,62)_=3.007, P=0.055), and brain region, exercise, and age (F_(2,62)_=2.029, P=0.139). *Post hoc* test revealed a significant increase in SOD activity in cerebellum of sedentary 18-month-old rats compared to brainstem of exercise 18-month-old rats (Figure 3B).

### Total thiol content

**Figure 4:**
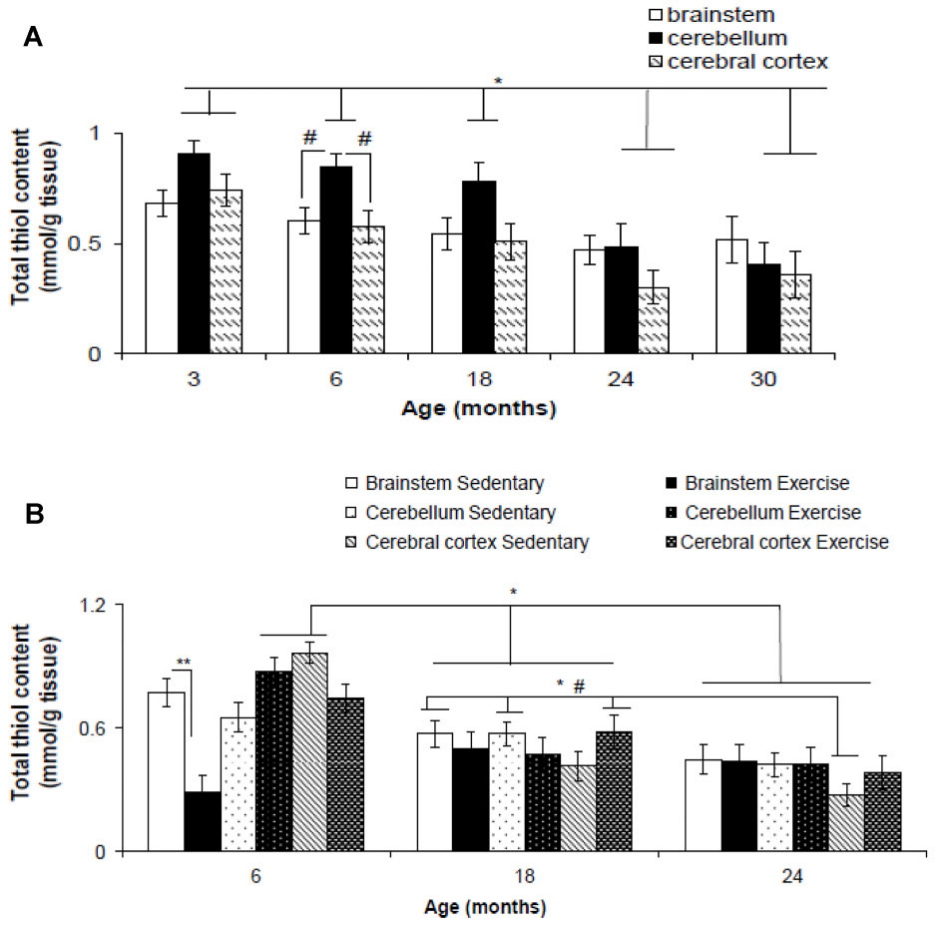
Total thiol content in the brainstem, cerebellum, and cerebral cortex of Wistar rats of different ages (**A**) and after 12 weeks of treadmill running (**B**). Data are reported as means±SE. *P<0.05, two- or three-way ANOVA; ^#^P<0.05 two- or three-way ANOVA; **P<0.05, three-way ANOVA.

A significant difference was found in total thiol content between age (F_(2,76)_=11.948, P<0.001) and brain region (F_(2,76)_=6.142, P=0.003) (Figure 4A). However, no significant interaction was found between the factors brain region and age (F_(2,76)_=1.056, P=0.403). *Post hoc* test revealed a significant decrease in total thiol content of the cerebellum and cerebral cortex at 24 and 30 months compared to 3 (P<0.001), 6 (P<0.001), and 18 (P=0.046) months, and cerebral cortex at 3 months (P<0.001). Total thiol content increased in cerebellum compared to brainstem (P=0.020) and cerebral cortex (P=0.020) at 6 months.

A significant difference was found for age (F_(2,62)_=30.332, P<0.001) but not for brain regions (F_(2,62)_=1.456, P=0.241) and exercise (F_(2,62)_=1.606, P=0.210). A significant interaction was found between factors brain region and age (F_(2,62)_=5.512, P<0.001) and brain region and exercise (F_(2,62)_=4.430, P=0.016). However, there was no significant interaction between factors age and exercise (F_(2,62)_=3.123, P=0.051). A significant interaction was found between factors brain region, exercise, and age (F_(2,62)_=5.125, P=0.001). *Post hoc* test revealed a significant decrease (P<0.001) in total thiol content in brainstem of exercise 3-month-old rats compared to this same region of sedentary rats (Figure 4B). Total thiol content increased in cerebellum of exercise and sedentary 3-month-old rats compared to brain regions of exercise and sedentary 18- and 24-month old rats (P=0.001). Total thiol content also increased in brainstem and cerebellum of sedentary 18-month-old rats compared to cerebral cortex of sedentary 24-month-old rats (P<0.001), and total thiol content increased in cerebral cortex of exercise 18-month-old rats compared to this same region of sedentary 24-month-old rats (P<0.001).

### Total antioxidant capacity

**Figure 5:**
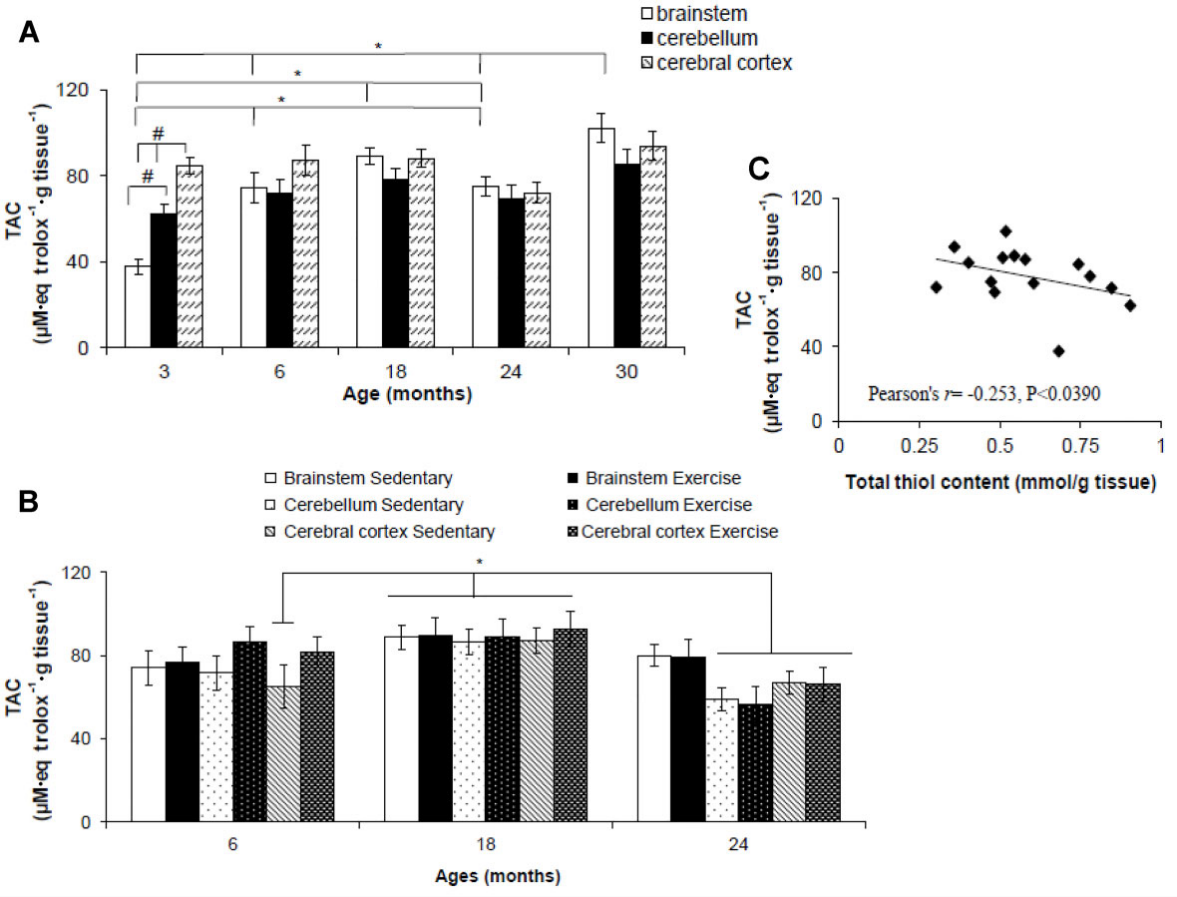
Total antioxidant capacity (TAC) (**A** and **B**) and correlation between TAC and total thiol content (**C**) in the brainstem, cerebellum, and cerebral cortex of Wistar rats of different ages (**A** and **C**) and after 12 weeks of treadmill running (**B**). Data are reported as means±SE. *P<0.05, two- or three-way ANOVA.

A significant difference was found for TAC between age (F_(2,76)_=21.410, P<0.001) but not brain region (F_(2,76)_=2.807, P=0.067). A significant interaction was found between the factors brain region and age (F_(2,76)_=8.036, P<0.001). *Post hoc* test revealed a significant decrease in TAC of the brainstem of 3-month-old rats compared to the brainstem of 6-, 18-, 24-, and 30-month-old rats (P=0.001) (Figure 5A). However, TAC increased in the brainstem of 18- and 30-month-old rats compared to 6- and 24-month-old rats (P=0.0042 and P<0.001, respectively). At 3 months, the lowest TAC levels were found in the brainstem. However, the highest levels were found in the cerebral cortex. In cerebellum, TAC showed intermediary values between brainstem and cerebral cortex.

A significant difference was found between age (F_(2,62)_=13.250, P<0.001) but not brain region (F_(2,62)_=1.235, P=0.298) and exercise (F_(2,62)_=1.511, P=0.224). No significant interaction was found between factors: brain region and age (F_(2,62)_=1.782, P=0.144), brain region and exercise (F_(2,62)_=0.246, P=0.783), exercise and age (F_(2,62)_=1.003, P=0.373), and brain region, exercise, and age (F_(2,62)_=0.168, P=0.954). *Post hoc* test revealed a significant increase in TAC in all brain regions of exercise and sedentary 18-month-old rats compared to cerebral cortex of sedentary 6-month-old rats (P=0.014) and cerebellum and cerebral cortex of exercise and sedentary 24-month-old rats (P<0.001) (Figure 5B).

There was a significant negative correlation between total thiol content and TAC in sedentary rats of different ages (r=-0.253, P<0.039; Figure 5C). No significant correlation was found between these markers after exercise (r=0.145, P=0.234).

### X-ray analysis of the femur

**Figure 6:**
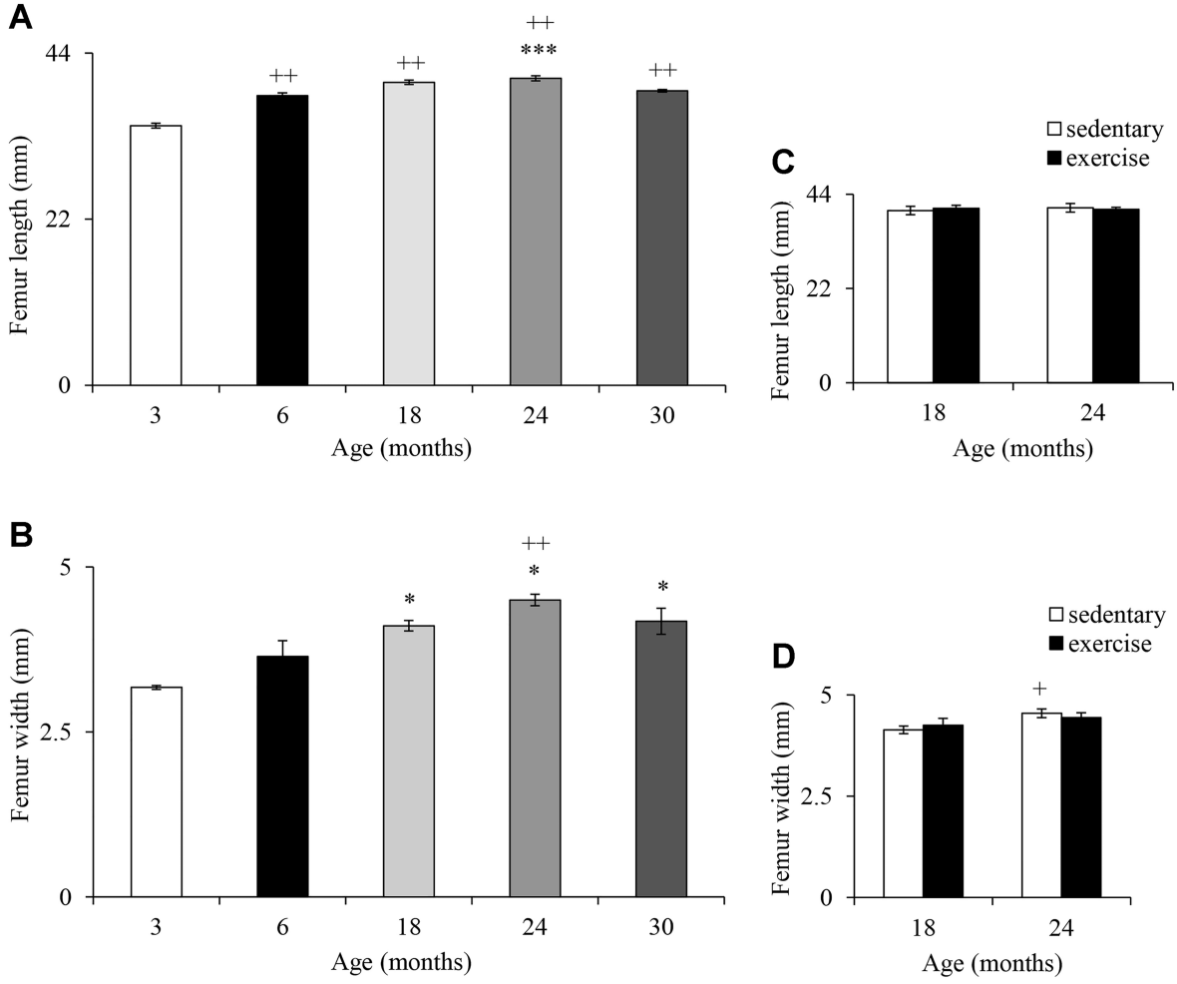
Femur length (**A**) and width (**B**) of Wistar rats of different ages and after 12 weeks of treadmill running (**C** and **D**). Data are reported as means±SE. *P<0.05 compared to 3- and 6-month-old rats; ***P<0.05 compared to 6-month-old rats; ^++^P<0.05 compared to 3-month-old rats; ^+^P<0.05 compared to 18-month-old sedentary rats [one- (**A** and **B**) and two-way (**C** and **D**) ANOVA followed by Tukey *post hoc* test].

The femur length was significantly greater (approximately 15%) for 6-, 18-, 24-, and 30-month-old rats compared to 3-month-old rats (Figure 6A). It was also significantly greater (6%) for 24-month-old rats compared to 6-month-old rats. Interestingly, femur length was similar between 30-month-old rats and 6-month-old rats, but shorter (4%) than in 18- and 24-month-old rats. Despite being insignificant, the femur width was 12% greater in 6-month-old than in 3-month-old rats. This measure was significantly greater (approximately 33%) in 18-, 24-, and 30-month-old rats than in 3-month-old rats (Figure 6B). The femur width was significantly greater in 24-month-old rats than in 6- and 18-month-old rats, being 25 and 9% greater at 24 months than at 6 and 18 months, respectively. Nevertheless, femur width was similar in 30-month-old rats and 18-month-old rats, but smaller (9%) than in 24-month-old rats. None of the parameters exhibited significant changes after low-intensity exercise (Figure 6C and D).

**Figure 7:**
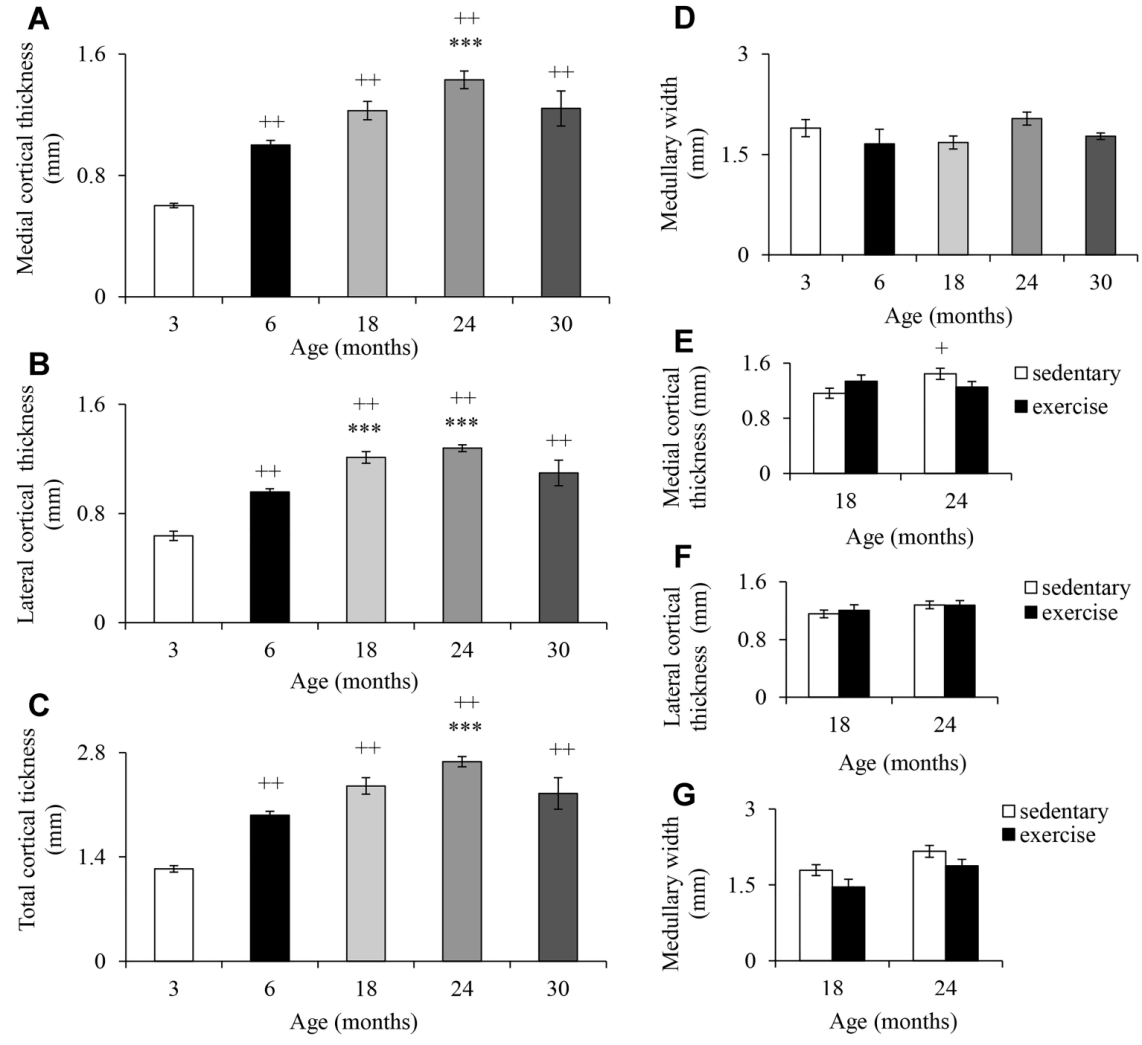
Medial (**A**) and lateral (**B**) cortical thickness, total cortical thickness (**C**), and medullary canal width (**D**) of the femur of Wistar rats of different ages and after 12 weeks of treadmill running (**E**-**G**). Data are reported as mean±SE. ^++^P<0.05 compared to 3-month-old rats; ***P<0.05 compared to 6-month-old rats; ^+^P<0.05 compared to 18-month-old sedentary rats [one- (**A**-**D**) and two-way (**F**-**G**) ANOVA followed by Tukey *post hoc* test].

Thickness of cortical bone in the middle third of the femur (lateral and medial) differed between ages. In the medial region, thickness was approximately 100% greater at 6, 18, 24, and 30 months than at 3 months (Figure 7A). This parameter was also significantly greater (40%) for 24-month-old rats compared to 6-month-old rats. Thickness of cortical bone in the medial region was similar in 30-month-old rats and 18-month-old rats, but 14% smaller than in 24-month-old rats. In the lateral region, the thickness was significantly greater (approximately 78%) at 6, 18, 24, and 30 months than at 3 months. Only 18- and 24-month-old rats had thickness of the lateral region significantly greater (approximately 30%) compared to 6-month-old rats (Figure 7B). The thickness of cortical bone in the lateral region was 14% smaller in 30-month-old than in 18- and 24-month-old rats. Similar results were found for total cortical thickness (medial+lateral) (Figure 7C). There was no significant change in medullary width (Figure 7D). None of the parameters exhibited significant change after low-intensity exercise (Figure 7E–G).

Table 1 summarizes other radiographic data. Aging induced sclerosis in the cortical bone and medullary cavity. In cortical bone, sclerosis was observed at 18, 24, and 30 months, being mild for 18-month-old rats and moderate for 24- and 30-month-old rats. In medullary cavity, sclerosis was observed only in 24- and 30-month-old rats, being severe in 24-month-old rats and moderate in 30-month-old rats. Severe radiolucency was observed in femur at 24 and 30 months. Only 30-month-old rats showed light radiopacity. Light bone deformity was observed at 18, 24, and 30 months of age. This change was located predominantly in greater trochanter or femoral condyles. Only one rat exhibited light deformity in lesser trochanter at 18 months. Remodeling of femoral neck was observed at 18 and 24 months, being moderate in 18-month-old rats and severe in 24-month-old rats. Fracture was not found in femur through aging. Osteophyte was found at 24 months. Regular low-intensity exercise did not change the occurrence of cortical sclerosis at 24 months, but it reduced this measure in medullary cavity. Exercise also reduced radiolucency at 24 months. However, exercise did not induce changes in the remodeling of femoral neck and bone deformity. None of the radiographic measures exhibited significant change in sedentary and exercise rats at 3 and 6 months.

**Table 1:** Radiographic measurements of femur from Wistar rats of different ages.

	Age (months)							
	Sedentary					Exercise		
	3	6	18	24	30	6	18	24
Sclerosis in cortical bone	-	-	+ (1/5)	++ (1/6)	++(1/3)	-	-	++ (2/4)
Sclerosis in medullar canal	-	-	-	+++ (3/6)	+ +(1/3)	-	-	-
Increased radiolucency	-	-	-	+++ (1/6)	+++(2/3)	-	-	++ (1/4)
Non-uniform radiopacity	-	-	-	-	+(1/3)	-	-	-
Bone deformity	-	-	++ (2/5)	++ (1/6)	+(1/3)	-	++ (1/3)	++ (1/4)
Remodeling of femoral neck	-	-	++ (3/5)	+++ (3/6)	-	-	++ (1/3)	+++ (2/4)
Fracture	-	-	-	-	-	-	-	-
Osteophyte	-	-	-	present (2/6)	-	-	-	-

Exercise: 12 weeks of treadmill running. (-): absent; (+): present light; (++) moderate; (+++) severe. The numbers in parenthesis represent the number of rats exhibiting the radiomorphological change/total number of radiographs from femur.

## Discussion

The present study showed the effects of aging and low intensity exercise on oxidative biomarkers in brainstem, cerebellum, and cerebral cortex. The effects of aging and exercise on femoral radiographic measurements were also evaluated. In brain regions, the lipid hydroperoxide levels increased in cerebellum compared to brainstem at 18 months. In a previous study, the levels of lipid peroxide and lipid hydroperoxide gradually increased in brainstem, cerebellum, and cerebrum of male albino rats at 6, 13, 19, and 24 months compared to 3 months (10). The difference between studies may be related to mitochondrial bioenergetics. Genetic, proteomic, or environmental changes have been shown to influence mitochondrial bioenergetics changes during aging (25). Nevertheless, lipid peroxides increased significantly in the cerebral cortex of 24-month-old Wistar rats compared to 3-month-olds (9), and in frontal and temporal cortices of 18-month-olds compared to 10-month-olds (26). In these studies, the quantification of lipid peroxidation products was performed based on the formation of substances reacting with thiobarbituric acid, or the final products of lipid peroxidation, e.g., malondialdehyde. According to Jiang et al. (1991), the assay involving thiobarbituric acid, in contrast to lipid hydroperoxide measurement by oxidation of Fe^2+^ in the presence of xylenol orange, does not measure hydroperoxides *per se*. This may explain the differences between studies. However, our study did not evaluate lipid hydroperoxides in specific cortical regions.

The drop in total thiol content during aging is in line with previous studies (7,9). However, our study adds novelty by showing that the drop is similar at 24 and 30 months. Since a reduction in total thiol content in lumbosacral spinal cord was observed in older rats (17), the decrease in total thiols seems to be a common event in the central nervous system of aged rats. Decreased total thiol content appears to be indicative of reduced efficiency of S-thiolation as a mechanism of antioxidant defense during aging (27). Thus, it may be suggested that this mechanism is reduced in the central nervous system of older Wistar rats.

While we found that TAC increased in the brainstem of old rats, in a previous study, this capacity reduced in the cerebral cortex, cerebellum, and brainstem of 24-month-old female Wistar rats compared to 3-month-olds (28). At present, we cannot explain this difference, but it may be suggested that the change was related to gender. Sex differences were found in nitric oxide levels and antioxidant defense systems in some regions of rat brain (29). Since TAC increased in brainstem (results of the present study) and lumbosacral spinal cord (17) of old rats, it may be suggested that changes in TAC differ in these regions compared to the higher ones of the neural axis.

Interestingly, thiols appear to be the major determinant of TAC in tissue homogenate (30). However, our study found significant negative correlation between changes in TAC and total thiols. According to Balcerczyk et al. (30), adaptation to oxidative stress may involve mobilization of mechanisms other than increase in thiols content. However, SOD activity did not vary significantly during aging. This suggests that other antioxidant systems could have their activity increased over aging. However, we have not evaluated other antioxidant systems. Future research will address this issue.

The lack of significant changes in SOD activity during aging has also been observed in other studies of rat brain up to 24 months (7,9). Thus, our study adds novelty by showing data from 30-month-old rats. As SOD is the enzyme that converts superoxide radicals into H_2_O_2_ (5), the maintenance of its activity indicates that this conversion remains unchanged during aging. However, while SOD activity increased significantly in lumbosacral spinal cord of old rats (17), it did not occur in the brain regions. Thus, the spinal cord seems to exhibit changes in SOD activity that differ from that of the brain during aging. In fact, differential age- and region-specific changes in rat brain mitochondrial function have been demonstrated (25). In addition, spinal cord mitochondria appear to produce more ROS and cause more oxidative damage than age-matched brain mitochondria (8).

Aging also caused changes in radiographic measurements of the femur. The increase in length and width of this bone is in accordance with literature data (24). In this study, the authors observed that the total bone mineral density of the femur increased up to 24 months, followed by a slight decrease at 30 months, and these changes occurred alongside changes in body weight. As it has been observed that body weight of Wistar rats increased up to 24 months, followed by a slight fall at 30 months (17), changes in femur length and width are likely related to changes in body weight.

The changes that occurred in the cortical bone and the lack of changes in the medullary cavity width was in line with previous studies (24,31,32). As the medullary cross-sectional area was significantly larger in older than young men, and this change seemed to indicate endosteal resorption (33), it may be suggested that this change did not occur in the femur of old rats. According to Iida and Fukuda (24), the cortical area decreased while the trabecular area increased with increasing age, which might be due to the dominance of periosteal formation rather than endosteal resorption by the modeling pattern in rats, indicating that the cortical thickness decreases with increasing age. In fact, radiolucency and remodeling of femoral neck increased in femur of old rats, which may be indicating a reduction in bone mass. The presence of cortical and medullary sclerosis may be related to inflammatory processes induced by aging. The production of tumor necrosis factor alpha and interleukin-6, pro-inflammatory cytokines, in the bone marrow mesenchymal stromal cells was significantly higher in the aged compared to adult rats (34). The lack of osteophyte in 30-month-old rats' femur may be related to the small number of animals. Taken together, our results suggested that femur exhibited the characteristic changes induced by aging.

The present study also demonstrated the effects of regular low-intensity exercise on brain oxidative parameters and radiographic measures of femur. The lack of significant changes in lipid hydroperoxide levels is in line with a previous study (16). According to those authors, the rodent training protocols between 20 and 30 min over a period of 8 weeks did not affect antioxidant enzymatic equilibrium, but aerobic exercise for more than 8 weeks induced an increase in catalase and SOD activities. In this context, the lack of significant changes in SOD activity may also be related to exercise intensity and/or frequency. Chalimoniuk et al. (35) observed that moderate intensity endurance training significantly enhances the number of components of the antioxidant barrier, including SOD activity, mostly in the neocortex, and to a lesser extent, in the cerebellum and striatum of adolescent Wistar rats.

The difference in exercise frequency and/or intensity may also be the cause of the lack of recovery of total thiols in different brain regions. In adolescent rats, endurance training for 13 weeks increased total glutathione content in cerebral cortex, cerebellum, and midbrain (35]. However, exercise training on a treadmill for 7.5 weeks did not significantly change the levels of glutathione in cortex, brainstem, and striatum of young rats (36). Interestingly, our study showed a significant decrease in total thiols level in brainstem of exercised 6-month-old rats. This finding may relate to different mechanisms triggered by exercise. It has been suggested that oxidative stress affects different pathways depending not only on the ROS/reactive nitrogen species quantity but also on their types, primary or secondary species (3).

However, exercise caused an improvement in radiographic measurements. There was a decrease in radiolucency and absence of medullary sclerosis in the femur of exercised rats. These results point towards the beneficial effect of exercise on aging-induced bone loss. However, a recent study showed that moderate but not low intensity exercise performed 4 times/week for 4 weeks returns the number of osteoclasts back to normal, which had been altered by d-galactose-induced aging (37). Since the difference between studies was exercise frequency and intensity, these results suggest that a beneficial effect of regular low-intensity exercise depends on its frequency and/or intensity.

However, our study had limitations. The number of rats per experimental group (5 or 6) was small. This weakened comparisons between groups and could result in false-negative findings. Also, we need to consider that the results may not be representative of the general population for 30-month-old rats (number of rats=3). Thus, further studies are necessary to clarify the effects of aging and low-intensity exercise on brain-oxidative biomarkers.

In conclusion, the rats of the present study experienced changes commonly found in brain-oxidative biomarkers and radiographic measurements of femur during aging. While data pointed towards the beneficial effect of low-intensity exercise on radiographic measurements of the femur, the changes were complex in oxidative biomarkers. Since the present study possessed limitations (small number of rats per group), a beneficial effect of regular low-intensity exercise on oxidative markers in brain cannot be ruled out.
